# Single vs Serial Measurements of Cardiac Troponin Level in the Evaluation of Patients in the Emergency Department With Suspected Acute Myocardial Infarction

**DOI:** 10.1001/jamanetworkopen.2020.37930

**Published:** 2021-02-23

**Authors:** Maereg Wassie, Ming-Sum Lee, Benjamin C. Sun, Yi-Lin Wu, Aileen S. Baecker, Rita F. Redberg, Maros Ferencik, Ernest Shen, Visanee Musigdilok, Adam L. Sharp

**Affiliations:** 1 Division of Cardiology, Kaiser Permanente Southern California, Los Angeles Medical Center, Los Angeles; 2 Department of Emergency Medicine, Leonard Davis Institute of Health Economics, University of Pennsylvania, Philadelphia; 3 Department of Research and Evaluation, Kaiser Permanente Southern California, Pasadena; 4 Division of Cardiology, University of California, San Francisco, San Francisco; 5 Editor, *JAMA Internal Medicine*; 6 Knight Cardiovascular Institute, Oregon Health and Science University, Portland

## Abstract

**Importance:**

Chest pain is among the most common reasons for emergency department (ED) presentations. However, most patients are at low risk for acute coronary syndrome (ACS), with low cardiac adverse outcomes rates. Biomarker testing with troponin levels is key in the initial assessment for ACS. Although serial troponin testing can improve the diagnosis of ACS in clinical practice, some patients deemed to be low risk are discharged after a single negative troponin test result.

**Objective:**

To report the clinical outcomes of patients discharged after a single negative troponin test result compared with patients discharged after serial troponin measurements.

**Design, Setting, and Participants:**

This is a retrospective cohort study of ED encounters from May 5, 2016, to December 1, 2017, across 15 community EDs within an integrated health care system in southern California. The study cohort includes 27 918 adult ED encounters in which patients were evaluated for suspected ACS with a HEART (history, electrocardiogram, age, risk factors, and troponin) score and an initial conventional troponin-I measurement below the level of detection (<0.02 ng/mL). Statistical analysis was performed from December 1, 2019, to December 1, 2020.

**Exposure:**

Single troponin test vs multiple troponin tests.

**Main Outcomes and Measures:**

The primary outcome was acute myocardial infarction or cardiac mortality; secondary outcomes included coronary artery bypass graft, percutaneous coronary intervention, invasive coronary angiography, and unstable angina within 30 days of discharge. A multivariable logistic regression model was performed to evaluate the association between testing strategies and clinical outcomes.

**Results:**

A total of 27 918 patient encounters (16 212 women [58.1%]; mean [SD] age, 58.7 [15.2] years) were included in the study. Of patients with an initial troponin measurement below the level of detection, 14 459 (51.8%) were discharged after a single troponin measurement, and 13 459 (48.2%) underwent serial troponin tests. After adjustment for cardiac risk factors and comorbidities, there was no statistically significant difference in the primary outcome of acute myocardial infarction or cardiac mortality within 30 days between the 2 groups (single troponin, 56 [0.4%] vs serial troponin, 52 [0.4%]; adjusted odds ratio, 1.41 [95% CI, 0.96-2.07]). Patients discharged after a single troponin test had lower rates of coronary artery bypass graft (adjusted odds ratio, 0.24 [95% CI, 0.11-0.48]) and invasive coronary angiography (adjusted odds ratio, 0.46 [95% CI, 0.38-0.56]).

**Conclusions and Relevance:**

This study suggests that patients are routinely discharged from the ED after a single negative troponin test result, and when compared with serial troponin testing, a single troponin test appears safe based on current physician decision-making, with no difference in rates of 30-day cardiac mortality and acute myocardial infarction, which are low in both groups.

## Key Points

**Question:**

Do patients discharged from the emergency department after a single troponin test with negative results have similar outcomes to patients undergoing multiple troponin tests?

**Findings:**

This cohort study found no significant difference in 30-day acute myocardial infarction or cardiac mortality between patients receiving a single troponin test with negative results and those who underwent serial troponin tests (single troponin test, 0.4% vs serial troponin tests, 0.4%).

**Meaning:**

This study suggests that physician discretion to order serial or single troponin tests in managing emergency department patients suspected to have acute coronary syndrome appears safe and is a reasonable strategy to possibly improve efficiency without an adverse association with patient outcomes.

## Teaser

This cohort study reports the clinical outcomes of patients discharged from the emergency department after a single negative troponin test result compared with those discharged after serial troponin measurements.

## Introduction

Chest pain and symptoms concerning for acute myocardial infarction (AMI) are among the most common reasons for emergency department (ED) presentation, accounting for more than 10 million ED visits annually.^1^ Biochemical testing and electrocardiography, along with clinical assessment, are cornerstones of the initial evaluation when a patient presents with chest pain and symptoms concerning for AMI. Prompt diagnosis is critical to initiate appropriate evidence-based therapies, while accurate and efficient exclusion of AMI is essential to avoid iatrogenic harms and for the efficient use of health care resources.

Current guidelines and several diagnostic algorithms recommend the repeated use of troponin tests and the detection of absolute change in the troponin level to safely rule out AMI in the ED.^2,3,4^ Recent studies measuring high-sensitivity troponin (hsTn) showed that, if the initial troponin level is very low, 1 troponin test with negative results may be sufficient to safely discharge a patient from the ED.^5,6,7^ These studies were performed using hsTn, but the use of hsTn is still low in the US (estimated at approximately 20% of US medical centers).^8^ In practice, some clinicians have adopted this strategy of a single troponin test using conventional troponin assays. The safety of this practice is not well studied, to our knowledge.

Several decision rules are available to help risk stratify patients presenting to the ED with symptoms concerning for AMI. The HEART (history, electrocardiogram, age, risk factors, and troponin) score is a validated risk stratification tool that incorporates clinical history with electrocardiography and troponin testing to help clinicians identify high-risk patients for further observation and low-risk patients who may be discharged and safely avoid further diagnostic testing.^9^ Incorporating the HEART score into clinical care pathways has been shown to reduce cardiac testing and length of stay and increase early discharges, while maintaining high sensitivity for detecting AMI.^10,11^ A single-center prospective cohort study that compared the performance of 3 troponin assays (high-sensitivity troponin T, high-sensitivity troponin I, and conventional troponin I) using the modified HEART score found that all 3 assays were equally effective in risk stratifying patients with chest pain for safe discharge, yielding a 100% sensitivity for 30-day mortality.^12^

Many patients who present to the ED have an initial troponin level that is in the undetectable range. The value of keeping low-risk patients with an initial undetectable troponin level in the ED for serial troponin measurements may be of marginal value. The goal of this study is to evaluate the safety of discharging patients with suspected AMI after a single negative conventional troponin test result (<0.02 ng/mL [to convert to micrograms per liter, multiply by 1.0], at a level below the limit of quantitation), incorporating the HEART score, compared with a strategy of serial troponin measurements for patients with suspected AMI.

## Methods

### Study Design and Population

We conducted a retrospective cohort study of prospectively collected data from 15 community EDs between May 5, 2016, and December 1, 2017, within Kaiser Permanente Southern California, an integrated health system with more than 4 million health plan members. Kaiser Permanente Southern California hospitals provide care to more than 1 million patients per year presenting to the ED. Of these ED visits, approximately 80% are by health plan members. Health plan members receive comprehensive services, including ambulatory, inpatient, pharmacy, imaging, and laboratory services. All care episodes are captured in a single integrated electronic health record among health plan facilities and through claims data for care outside of the integrated system. This study was approved by the Kaiser Permanente Southern California Institutional Review Board, which waived the need for informed consent given the retrospective nature of this observational study that involved no direct patient contact to evaluate the impact of a quality improvement initiative to optimize care for patients evaluated in the ED. Our study followed the Strengthening the Reporting of Observational Studies in Epidemiology (STROBE) reporting guideline.^13^

### Patient Cohort

The study cohort includes ED encounters in which an adult was evaluated for chest pain suspected for acute coronary syndrome (ACS) with a HEART score and an initial conventional troponin I level below the limit of quantification (<0.02 ng/mL). The patients included were with Kaiser Permanente Southern California health plan membership of at least 12 months prior to their ED visit (allowing a 30-day gap). Patients who were nonmembers or did not have continuous insurance coverage for 1 year prior to their ED visit were excluded because we do not have accurate comorbidity data or follow-up information for their outcomes. Patients with a do-not-resuscitate status or hospice status were also excluded.

### Troponin Measurements

All sites used the same conventional troponin I assay (Access AccuTnI+3 assay; Beckman-Coulter). The limit of quantitation is 0.02 ng/mL. The 99th percentile is 0.04 mg/mL. The coefficient of variation at a concentration of 0.04 ng/mL is 10%. The coefficient for variation at a concentration of 0.02 ng/mL is 20%.^14^

### Covariate Measurements

Baseline demographic information, including age, sex, and race/ethnicity data, were obtained from administrative records. Our administrative data were previously shown to be accurate in describing race/ethnicity.^15^ Clinical patient variables were obtained by querying the structured electronic health records. The *International Classification of Diseases, Ninth Revision* (*ICD-9*) and *International Statistical Classification of Diseases and Related Health Problems, Tenth Revision* (*ICD-10*) codes used to define the clinical variables (such as hypertension, diabetes, dyslipidemia, coronary artery disease, stroke, percutaneous coronary intervention, and coronary artery bypass graft) can be found in the eAppendix in the Supplement. Body mass index was measured from ED intake documentation or the most recently available visit. Smoking and family history of coronary artery disease were self-reported in electronic health records. Those with a history of percutaneous coronary intervention or coronary artery bypass graft were considered to have had prior coronary revascularization.

### HEART Score

The HEART score was calculated using clinical and demographic data. HEART is a 0- to 10-point scoring system initially developed in the Netherlands and validated in Europe,^16,17^ as well as the US,^18,19^ and recommended by the American Heart Association.^9^ Scores are categorized as low risk (0-3), moderate risk (4-6), and high risk (7-10) to aid clinical decision-making. Details about the implementation of the HEART score in May 2016 at the participating study sites has been previously reported.^20,21^ At the time of the implementation of the HEART score, data on physician education were provided to encourage repeated troponin testing at least 3 hours after symptom onset or after the peak of symptom severity. Physician education was provided via a prerecorded continuing education module recommending physicians order repeated troponin tests for patients with symptoms for less than 3 hours. Physicians were encouraged to use timing of symptoms, initial troponin value, and initial HEART score to assess the benefit associated with repeated troponin testing and further clinical management. The data for the timing of the onset of chest pain are not available for this study.

### Outcome Measures

The primary outcome was 30-day cardiac mortality or myocardial infarction after ED discharge. Secondary outcomes were unstable angina, invasive coronary angiography, percutaneous transluminal coronary angioplasty, or coronary artery bypass graft.

Myocardial infarction was considered to have occurred if an *ICD-9* or *ICD-10* code for myocardial infarction was the principal diagnosis listed at a subsequent inpatient or ED encounter either within the integrated health system or from claims for services provided at an out-of-network facility. Mortality was assessed using a death registry that included data obtained from the California death index, the federal Social Security death index, and membership files. Cardiac or noncardiac cause of death was analyzed by a physician (M.W.) reviewing the stated cause of death in *ICD-9* and *ICD-10* codes and California state records.

### Statistical Analysis

Statistical analysis was performed from December 1, 2019, to December 1, 2020. Baseline characteristics are summarized using descriptive statistics. A comparison between the group undergoing a single troponin test and the group undergoing serial troponin tests was made by conducting *t* tests (for normally distributed continuous variables) or Wilcoxon rank sum tests when appropriate. Categorical variables were compared using the χ^2^ test. Multivariable logistic regression models were conducted to investigate the association between a strategy of serial troponin measurements and cardiovascular outcomes. Separate analyses were conducted for each cardiac outcome (AMI, unstable angina, and revascularization) and cardiac mortality. Analyses were performed using SAS, version 9.3 (SAS Institute Inc). Reported *P* values are 2-sided, and the level of statistical significance was *P* < .05.

## Results

### Baseline Characteristics

A total of 27 918 patient encounters (16 212 women [58.1%]; mean [SD] age, 58.7 [15.2] years) were included in the study (Table 1). Between May 5, 2016, and December 1, 2017, there were a total of 37 250 ED encounters with a troponin test order and HEART score (Figure). Among these patients, 6618 (17.8%) had an initial troponin level of 0.02 ng/mL or more and were excluded from the analysis. After application of the inclusion and exclusion criteria, 27 918 patients were included in the final analysis. A total of 14 459 patients (51.8%) had a single troponin test ordered, and a total of 13 459 patients (48.2%) had serial troponin tests ordered.

**Table 1.** Baseline Characteristics, Comorbidities, and Risk Factors for Patients With a First Troponin Level of Less Than 0.02 ng/mL

Characteristic	Troponin test orders, No. (%)		*P* value
	1 (n = 14 459)	≥2 (n = 13 459)	
Age, mean (SD), y	57.1 (16.1)	60.4 (13.9)	<.001
Female	8617 (59.6)	7595 (56.4)	<.001
Race/ethnicity			
White	5227 (36.2)	5047 (37.5)	<.001
Black	2198 (15.2)	2061 (15.3)	
Hispanic	5380 (37.2)	4701 (34.9)	
Asian or Pacific Islander	1231 (8.5)	1258 (9.3)	
Other^a^	423 (2.9)	392 (2.9)	
Smoking behavior			
Never	8739 (60.4)	8077 (60.0)	<.001
Quit	4158 (28.8)	4133 (30.7)	
Passive	82 (0.6)	55 (0.4)	
Active	1033 (7.1)	887 (6.6)	
Missing	447 (3.1)	307 (2.3)	
Admitted to hospital	977 (6.8)	2552 (19.0)	<.001
Elixhauser comorbidities, mean (SD), No.	3.6 (2.92)	3.8 (2.83)	<.001
Congestive heart failure	916 (6.3)	999 (7.4)	<.001
Cardiac arrhythmia	2316 (16.0)	2477 (18.4)	<.001
Valvular disease	658 (4.6)	681 (5.1)	.047
Type 1 and 2 diabetes	3746 (25.9)	4083 (30.3)	<.001
Kidney failure	1781 (12.3)	1945 (14.5)	<.001
Liver disease	1397 (9.7)	1365 (10.1)	.18
Obesity	5910 (40.9)	6059 (45.0)	<.001
Coronary artery disease	1793 (12.4)	2784 (20.7)	<.001
Stroke	416 (2.9)	391 (2.9)	.89
Lipid disorder	8188 (56.6)	8959 (66.6)	<.001
Medications (90 d before or after ED visit)			
Diabetes medication	2672 (18.5)	2946 (21.9)	<.001
Anticoagulant medication	1753 (12.1)	2270 (16.9)	<.001
Antihypertensive medication	7531 (52.1)	8316 (61.8)	<.001
ACE inhibitor or ARB	4646 (32.1)	5412 (40.2)	
Aldosterone receptor blocker	296 (2.0)	270 (2.0)	.81
β-Blocker	3745 (25.9)	4527 (33.6)	<.001
Calcium channel blocker	2314 (16.0)	2541 (18.9)	<.001
Diuretic	3337 (23.1)	3475 (25.8)	<.001
Statin	5389 (37.3)	6572 (48.8)	<.001

Abbreviations: ACE, angiotensin-converting enzyme; ARB, angiotensin receptor blocker; ED, emergency department.

SI conversion factor: To convert troponin to micrograms per liter, multiply by 1.0.

^a^ All races/ethnicities that were not specified as White, Black, Hispanic, Asian, or Pacific Islander.

**Figure.**
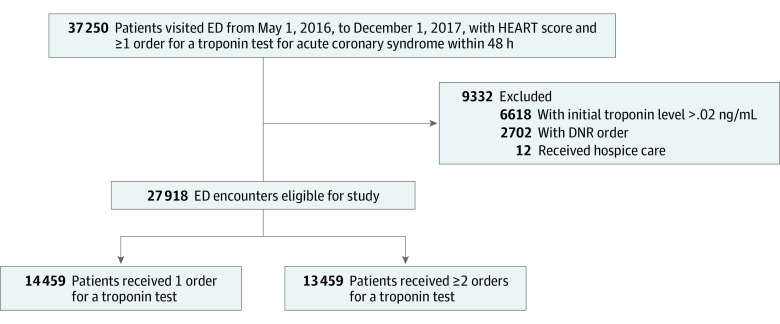
Flowchart With Inclusion and Exclusion Criteria DNR indicates do not rescusitate; ED, emergency department; and HEART, history, electrocardiogram, age, risk factors, and troponin. SI conversion factor: To convert troponin to micrograms per liter, multiply by 1.0.

The mean (SD) age of patients who underwent a single troponin test was 57.1 (16.1) years, and 8617 (59.6%) were women (Table 1). Patients discharged from the ED after a single troponin test indicating a level of less than 0.02 ng/mL were younger than those who underwent serial troponin tests and were less likely to have comorbid conditions, including hypertension, diabetes, lipid disorder, kidney failure, and congestive heart failure.

Of 27 918 patients with an initial negative troponin test result, 14 459 (51.8%) were discharged after a single troponin test, 10 611 (38.0%) had 2 troponin tests performed, and 2848 (10.2%) had 3 or more serial troponin tests performed (Table 2). Among those who underwent repeated troponin testing, most of the repeated tests were ordered 3 to 6 hours after the first troponin test was performed (Table 3). Among patients who did undergo repeated troponin testing, very few (66 [0.5%]) had a positive test result (troponin level >0.5 ng/mL).

**Table 2.** Data on Troponin Tests Ordered Within 48 Hours Among Patients With a First Troponin Level of Less Than 0.02 ng/mL, Stratified by HEART Score^a^

HEART score	Total tests (N = 27 918)	Troponin test orders, No. (%)		*P* value
		1 (n = 14 459)	≥2 (n = 13 459)	
0	1973 (7.1)	1587 (11.0)	386 (2.9)	<.001
1	3557 (12.7)	2448 (16.9)	1109 (8.2)	
2	5935 (21.3)	3535 (24.4)	2400 (17.8)	
3	6264 (22.4)	3342 (23.1)	2922 (21.7)	
4	5201 (18.6)	2283 (15.8)	2918 (21.7)	
5	3053 (10.9)	901 (6.2)	2152 (16.0)	
6	1438 (5.2)	280 (1.9)	1158 (8.6)	
7	439 (1.6)	70 (0.5)	369 (2.7)	
8	58 (0.2)	13 (0.1)	45 (0.3)	

Abbreviation: HEART, history, electrocardiogram, age, risk factors, and troponin.

SI conversion factor: To convert troponin to micrograms per liter, multiply by 1.0.

^a^ Time 0 = time of first troponin test.

**Table 3.** Data on Patients With Repeated Troponin Tests at Various Time Points During First 48 Hours if Troponin Level Is Less Than 0.02 ng/mL With First Test^a^

Time	No. (%) (N = 27 918)
1 Order only	14 459 (51.8)
0-1 h	100 (0.4)
1-2 h	662 (2.4)
2-3 h	3147 (11.3)
3-6 h	7462 (26.7)
6-9 h	1610 (5.8)
9-12 h	260 (0.9)
12-18 h	137 (0.5)
18-24 h	45 (0.2)
≥24 h	36 (0.1)

SI conversion factor: To convert troponin to micrograms per liter, multiply by 1.0.

^a^ Time 0 = time of first troponin test. If there were multiple troponin values after the first one, we used the maximum value for the summary tables. Hour calculation is based on the specimen collection time.

Follow-up information was available for 25 702 (92.1%) of patients. Table 4 shows the clinical outcomes. Overall, the rates of AMI and cardiac mortality were low during 30 days of follow-up (AMI, 95 of 27 918 [0.3%]; cardiac mortality, 13 of 27 918 [0.05%]). Compared with patients who underwent serial troponin testing, patients discharged after a single negative troponin test result did not have a statistically significant difference between risk of AMI or cardiac mortality at 30 days (single troponin test, 56 [0.4%] vs serial troponin tests, 52 [0.4%]; adjusted odds ratio [OR], 1.41 [95% CI, 0.96-2.07]). When broken down into individual components, there was also no statistically significant difference in the risk of AMI (OR, 1.28 [95% CI, 0.85-1.92]) or cardiac mortality (OR, 3.02 [95% CI, 0.97-12.08]) between the 2 groups, with the serial troponin test group as the reference.

**Table 4.** Outcomes at 30 Days

Outcome	Troponin test orders, No. (%)		Odds ratio (95% CI)^a^	Crude odds ratio (95% CI)
	1 (n = 14 459)	≥2 (n = 13 459)		
Cardiac mortality or myocardial infarction	56 (0.4)	52 (0.4)	1.41 (0.96-2.07)	1.00 (0.69-1.46)
Cardiac mortality	10 (0.07)	3 (0.02)	3.02 (0.97-12.08)	2.79 (0.91-11.07)
Myocardial infarction	46 (0.3)	49 (0.4)	1.28 (0.85-1.92)	0.87 (0.58-1.31)
Coronary artery bypass graft	8 (0.06)	51 (0.4)	0.24 (0.11-0.48)	0.15 (0.07-0.30)
Percutaneous coronary intervention	24 (0.2)	47 (0.3)	0.73 (0.44-1.18)	0.48 (0.29-0.77)
Invasive coronary angiography	138 (1.0)	380 (2.8)	0.46 (0.38-0.56)	0.33 (0.27-0.40)
Unstable angina	69 (0.5)	87 (0.6)	1.05 (0.76-1.44)	0.74 (0.54-1.01)

^a^ Logistic regression model adjusted for age, sex, hypertension, hyperlipidemia, diabetes, smoking, obesity, prior history of coronary artery disease, and Elixhauser comorbidity index.

In analysis of secondary outcomes, patients discharged after a single negative troponin test result had lower adjusted rates of coronary artery bypass graft (OR, 0.24 [95% CI, 0.11-0.48]) and invasive coronary angiogram (OR, 0.46 [95% CI, 0.38-0.56]). However, there was no statistically significant difference between the groups in the rates of percutaneous coronary intervention (OR, 0.73, [95% CI, 0.44-1.18]) and unstable angina (OR, 1.05 [95% CI, 0.76-1.44]) (Table 4).

## Discussion

In this large retrospective cohort study analyzing the safety of discharging patients after a single nondetectable conventional troponin level, we found that use of a single troponin measurement, when deemed appropriate by the clinician caring for the patient, was safe and not associated with differences in 30-day AMI or cardiac mortality. We also observed that patients discharged after a single troponin test had a lower rate of coronary artery bypass graft and a lower rate of invasive coronary angiography within 30 days.

The American Heart Association and European Society of Cardiology guidelines recommend serial cardiac troponin tests to identify and exclude myocardial infarction as well as to assess the acuity of a cardiac event in an individual with suspected ACS.^2,3,4^ Serial testing has been recommended by most to safely rule out AMI. Elevated troponin levels can be detected as early as 2 to 4 hours after the onset of symptoms and can be delayed for up to 8 to 12 hours. Several early studies have demonstrated that the slow increase and late peak of troponin levels make the conventional troponin assay vulnerable to missing early evolving AMI.^22,23,24^ However, most patients presenting to the ED with chest pain are at low risk for ACS and are below a 1% risk threshold of 30-day adverse events.^18^ Therefore, there may not be a benefit associated with repeated troponin testing for low-risk patients and those with prolonged symptoms, despite guideline recommendations. In our cohort, 51.8% of patients with an initial troponin evaluation did not undergo repeated troponin testing. In addition, among patients who did undergo repeated troponin testing, very few (66 [0.5%]) had a positive test result (troponin level >0.5 ng/mL). Our results also demonstrate that, in clinical practice, the strategy of administering a single troponin test is safe when the test is performed along with a clinician’s clinical assessment and risk stratification by the HEART score.

Many experts agree that there is broad overuse of troponin tests for clinical presentations even when the suspicion for AMI is low.^25^ A previous multicenter study, in which 64% of troponin measurements were single assesssments, found that 79% of elevated troponin levels were associated with diagnoses other than AMI.^26^ When the troponin test was first introduced for clinical use, it was thought to be specific for AMI because the population in which it was studied had a significantly higher prevalence of coronary artery disease. However, its current use in the general population and with hsTn has made it less specific for AMI when results are positive. In a prospective cohort study of patients presenting to the ED in the UK and US, only 4.2% had an AMI.^25,27^ Our study demonstrates that physicans are able to appropriately identify patients at higher risk based on other clinical factors beyond the results of a second troponin test.

Furthermore, the ability to effectively and promptly rule out AMI using results of a single troponin test can reduce the need to keep patients in the ED for serial troponin testing. Overcrowding of the ED and increased hospital length of stay are a major health care concern. Crowding of the ED is associated with patient outcomes and the quality of care provided.^28^ Chest pain evaluation accounts for a substantial health care cost, representing approximately 7 million yearly ED visits, with an estimated cost of $5 billion.^29^ Strategies allowing rapid and accurate discharge of low-risk patients after a single troponin test can potentially reduce cost and allow resources to be allocated for other patients in need.

There was a statistically insignificant difference in cardiac mortality and myocardial infarction between the group of patients who underwent a single troponin test and the group of patients who underwent serial troponin tests, with both groups having very low 30-day cardiovascular outcomes. A survey of ED physicians found the tolerable rate of missed major adverse cardiac events to be less than 1%.^30^ An analysis by Kline et al^31^ showed that a 2% rate of missed diagnoses of ACS might be considered acceptable considering the risk of harm from further testing. Many physicians are currently using the strategy of a single troponin test, and it appears to be as accurate and safe as serial troponin tests as a diagnostic approach for selected groups of patients. This strategy can prove useful in situations of high health care demand owing to increasing costs, ED crowding, or a limited resource setting, such as the coronavirus disease 2019 pandemic.

This study was performed with the conventional troponin assay. Early studies with hsTn (hsTn level <5 ng/L) show high accuracy for a rapid rule out of patients in the ED.^5,8,32^ Compared with the conventional troponin assay, the hsTn assay has higher sensitivity with a more pronounced difference in performance for patients presenting with recent-onset chest pain,^22^ as well as for those with low to moderate risk.^33^ The imprecision of the conventional troponin assay is indicated by its high coefficient of variation of −20% at a level of 0.02 ng/mL.^14,34^ Given the lower performance of the conventional troponin assay, supplementation with risk-stratification tools, such as the HEART and Emergency Department Assessment of Chest Pain scores, may improve the accuracy of the use of a single troponin test to rule out ACS.^14^ This approach can improve the patient experience by decreasing the length of stay in the ED and help to relieve ED overcrowding while maintaining safety.

### Strengths and Limitations

This study has several strengths. It is a large population-based study that reflects the practice pattern in multiple community-based EDs across southern California. The study population is diverse, and follow-up information is available for all patients. Cohort members have comprehensive health insurance, thereby minimizing selection bias.

Some limitations also need to be considered in the interpretation of the findings. There are no data on the timing of symptom onset because it is highly associated with the clinician’s decision-making. Given the observational study design, causality cannot be assumed. Residual confounding may be present despite adjustments of multiple variables. The data set used in this analysis lacks the detail available in trials and registries; particularly, we cannot assess the timing of symptom onset, which may be the key variable in determining which patients are most likely to experience a benefit associated with serial troponin testing.

## Conclusions

The findings of this cohort study suggest that, when compared with serial troponin testing, physician discretion to safely discharge a patient after an undetectable troponin level using a conventional troponin test, together with the HEART score, is a safe and reliable strategy for patients with suspected ACS (no difference in rates of 30-day cardiac mortality and AMI). Especially as results of hsTn assays become available, clinical recommendations should consider the physician’s discretion to use a single troponin test, which may help to improve resource allocation.

## Notes

Supplement. **eAppendix.** ICD-9-CM, ICD-10-CM, and CPT Diagnosis and Procedure Codes Used for Acute Myocardial Infarction (AMI), Coronary Artery Bypass Grafting (CABG), Coronary Artery Disease (CAD), Dyslipidemia, Stroke, and Percutaneous Coronary Intervention (PCI)
