# Supplementary material for: Single vs Serial Measurements of Cardiac Troponin Level in the Evaluation of Patients in the Emergency Department With Suspected Acute Myocardial Infarction
Source: JAMA Netw Open. 2021 Feb 23;4(2):e2037930. doi: 10.1001/jamanetworkopen.2020.37930 (PMC7903256; doi:10.1001/jamanetworkopen.2020.37930)
Supplement: Supplement. — eAppendix. ICD-9-CM, ICD-10-CM, and CPT Diagnosis and Procedure Codes Used for Acute Myocardial Infarction (AMI), Coronary Artery Bypass Grafting (CABG), Coronary Artery Disease (CAD), Dyslipidemia, Stroke, and Percutaneous Coronary Intervention (PCI) [file jamanetwopen-e2037930-s001.pdf]

Supplementary Online Content

Wassie M, Lee MS, Sun BC, et al. Single vs serial measurements of cardiac troponin level in the evaluation of patients in the emergency department with suspected acute myocardial infarction. *JAMA Netw Open.* 2021;4(2):e2037930. doi:10.1001/jamanetworkopen.2020.37930

eAppendix. ICD-9-CM, ICD-10-CM, and CPT Diagnosis and Procedure Codes Used for Acute Myocardial Infarction (AMI), Coronary Artery Bypass Grafting (CABG), Coronary Artery Disease (CAD), Dyslipidemia, Stroke, and Percutaneous Coronary Intervention (PCI)

This supplementary material has been provided by the authors to give readers additional information about their work.

© 2021 Wassie M et al. *JAMA Network Open.*

eAppendix. ICD-9-CM, ICD-10-CM, and CPT Diagnosis and Procedure Codes Used for Acute Myocardial Infarction (AMI), Coronary Artery Bypass Grafting (CABG), Coronary Artery Disease (CAD), Dyslipidemia, Stroke, and Percutaneous Coronary Intervention (PCI)

<u>AMI ICD-9-CM Codes:</u> 410.00, 410.0, 1410.01, 410.02, 410.10, 410.11, 410.11, 410.11, 410.11, 410.12, 410.20, 410.21, 410.21, 410.22, 410.30, 410.31, 410.31, 410.32, 410.40, 410.41, 410.41, 410.42, 410.50, 410.51, 410.51, 410.52, 410.60, 410.61, 410.61, 410.62, 410.70, 410.71, 410.71, 410.72, 410.80, 410.81, 410.81, 410.81, 410.82, 410.90, 410.91, 410.91, 410.92

<u>AMI ICD-10-CM Codes:</u> I21, I22

<u>CABG ICD-9-CM Codes:</u> 36.1-36.17, 36.19, 36.2, 36.3, 36.31-36.34, 36.39

<u>CABG ICD-10-CM Codes:</u> 0210083, 0210088, 0210089, 021008C, 021008F, 021008W, 0210093, 0210098, 0210099, 021009C, 021009F, 021009W, 02100A3, 02100A8, 02100A9, 02100AC, 02100AF, 02100AW, 02100J3, 02100J8, 02100J9, 02100JC, 02100JF, 02100JW, 02100K3, 02100K8, 02100K9, 02100KC, 02100KF, 02100KW, 02100Z3, 02100Z8, 02100Z9, 02100ZC, 02100ZF, 0210344, 02103D4, 0210444, 0210483, 0210488, 0210489, 021048C, 021048F, 021048W, 0210493, 0210498, 0210499, 021049C, 021049F, 021049W, 02104A3, 02104A8, 02104A9, 02104AC, 02104AF, 02104AW, 02104D4, 02104J3, 02104J8, 02104J9, 02104JC, 02104JF, 02104JW, 02104K3, 02104K8, 02104K9, 02104KC, 02104KF, 02104KW, 02104Z3, 02104Z8, 02104Z9, 02104ZC, 02104ZF, 0211083, 0211088, 0211089, 021108C, 021108F, 021108W, 0211093, 0211098, 0211099, 021109C, 021109F, 021109W, 02110A3, 02110A8, 02110A9,

© 2021 Wassie M et al. *JAMA Network Open.*

02110AC, 02110AF, 02110AW, 02110J3, 02110J8, 02110J9, 02110JC, 02110JF, 02110JW, 02110K3, 02110K8, 02110K9, 02110KC, 02110KF, 02110KW, 02110Z3, 02110Z8, 02110Z9, 02110ZC, 02110ZF, 0211344, 02113D4, 0211444, 0211483, 0211488, 0211489, 021148C, 021148F, 021148W, 0211493, 0211498, 0211499, 021149C, 021149F, 021149W, 02114A3, 02114A8, 02114A9, 02114AC, 02114AF, 02114AW, 02114D4, 02114J3, 02114J8, 02114J9, 02114JC, 02114JF, 02114JW, 02114K3, 02114K8,02114K9, 02114KC, 02114KF, 02114KW, 02114Z3, 02114Z8, 02114Z9, 02114ZC, 02114ZF, 0212083, 0212088, 0212089, 021208C, 021208F, 021208W, 0212093, 0212098, 0212099, 021209C, 021209F, 021209W, 02120A3, 02120A8, 02120A9, 02120AC, 02120AF, 02120AW, 02120J3, 02120J8, 02120J9, 02120JC, 02120JF, 02120JW, 02120K3, 02120K8, 02120K9, 02120KC, 02120KF, 02120KW, 02120Z3, 02120Z8, 02120Z9, 02120ZC, 02120ZF, 0212344, 02123D4, 0212444, 0212483, 0212488, 0212489, 021248C, 021248F, 021248W, 0212493, 0212498, 0212499, 021249C, 021249F, 021249W, 02124A3, 02124A8, 02124A9, 02124AC, 02124AF, 02124AW, 02124D4, 02124J3, 02124J8, 02124J9, 02124JC, 02124JF, 02124JW, 02124K3, 02124K8, 02124K9, 02124KC, 02124KF, 02124KW, 02124Z3, 02124Z8, 02124Z9, 02124ZC, 02124ZF, 0213083, 0213088, 0213089, 021308C, 021308F, 021308W, 0213093, 0213098, 0213099, 021309C, 021309F, 021309W, 02130A3, 02130A8, 02130A9, 02130AC, 02130AF, 02130AW, 02130J3, 02130J8, 02130J9, 02130JC, 02130JF, 02130JW, 02130K3, 02130K8, 02130K9, 02130KC, 02130KF, 02130KW, 02130Z3, 02130Z8, 02130Z9, 02130ZC, 02130ZF, 0213344, 02133D4, 0213444, 0213483, 0213488, 0213489, 021348C, 021348F, 021348W, 0213493, 0213498, 0213499, 021349C, 021349F,

© 2021 Wassie M et al. *JAMA Network Open.*

021349W, 02134A3, 02134A8, 02134A9, 02134AC, 02134AF, 02134AW, 02134D4, 02134J3, 02134J8, 02134J9, 02134JC, 02134JF, 02134JW, 02134K3, 02134K8, 02134K9, 02134KC, 02134KF, 02134KW, 02134Z3, 02134Z8, 02134Z9, 02134ZC, 02134ZF

CAD ICD-9-CM Codes: 410.0, 410.00, 410.01, 410.02, 410.1, 410.10, 410.11, 410.12, 410.2, 410.20, 410.21, 410.22, 410.3, 410.30, 410.31, 410.32, 410.4, 410.40, 410.41, 410.42, 410.5, 410.50, 410.51, 410.52, 410.6, 410.60, 410.61, 410.62, 410.7, 410.70, 410.71, 410.72, 410.8, 410.80, 410.81, 410.82, 410.9, 410.90, 410.91, 410.92, 411.0, 411.1, 411.8, 411.81, 411.89, 412, 413.0, 413.1, 413.9, 414.0, 414.00, 414.01, 414.06, 414.2, 414.3, 414.4, 414.8, 414.9, V45.81, V45.82

CAD ICD-10-CM Codes: I2101, I2102, I2109, I2111, I2119, I2121, I2129, I213, I214, I219, I21A1, I21A9, I220, I221, I222, I228, I229, I200, I201, I208, I209, I237, I240, I241, I248, I249, I2510, I25110, I25111, I25118, I25119, I252, I255, I256, I25750, I25751, I25758, I25759, I25811, I2582, I2583, I2584, I2589, I259, Z951, Z955, Z9861

Chest Pain ICD-9-CM Codes: 413.XX, 786.5X

Chest Pain ICD-10-CM Codes: I20.1, I20.8, I20.9, I25.111, I25.118, I25.119, I25.701, I25.708, I25.709, I25.711, I25.718, I25.719, I25.721, I25.728, I25.729, I25.731, I25.738, I25.739, I25.751, I25.758, I25.759, I25.761, I25.768, I25.769, I25.791, I25.798, I25.799, R07.9, R07.2, R07.1, R07.81, R07.82, R07.89

Dyslipidemia ICD-9-CM Codes: 2720, 2721, 2722, 2723, 2724

Dyslipidemia ICD-10-CM Codes: E780, E7800, E7801, E781, E782, E783, E784, E785

© 2021 Wassie M et al. *JAMA Network Open.*

Stroke ICD-9-CM Codes: 34660, 34661, 34662, 34663, 430, 431, 4320, 4321, 4329, 43301, 43311, 43321, 43331, 43381, 43391, 4340, 43400, 43401, 4341, 43410, 43411, 4349, 43490, 43491, 436

Stroke ICD-10-CM Codes: G43601, G43609, G43611, G43619, I6000, I6001, I6002, I6010, I6011, I6012, I602, I6020, I6021, I6022, I6030, I6031, I6032, I604, I6050, I6051, I6052, I606, I607, I608, I609, I610, I611, I612, I613, I614, I615, I616, I618, I619, I6200, I6201, I6202, I6203, I621, I629, I6300, I63011, I63012, I63013, I63019, I6302, I63031, I63032, I63033, I63039, I6309, I6310, I63111, I63112, I63113, I63119, I6312, I63131, I63132, I63133, I63139, I6319, I6320, I63211, I63212, I63213, I63219, I6322, I63231, I63232, I63233, I63239, I6329, I6330, I63311, I63312, I63313, I63319, I63321, I63322, I63323, I63329, I63331, I63332, I63333, I63339, I63341, I63342, I63343, I63349, I6339, I6340, I63411, I63412, I63413, I63419, I63421, I63422, I63423, I63429, I63431, I63432, I63433, I63439, I63441, I63442, I63443, I63449, I6349, I6350, I63511, I63512, I63513, I63519, I63521, I63522, I63523, I63529, I63531, I63532, I63533, I63539, I63541, I63542, I63543, I63549, I6359, I636, I638, I639, I6601, I6602, I6603, I6609, I6611, I6612, I6613, I6619, I6621, I6622, I6623, I6629, I663, I668, I669, R29700, R29701, R29702, R29703, R29704, R29705, R29706, R29707, R29708, R29709, R29710, R29711, R29712, R29713, R29714, R29715, R29716, R29717, R29718, R29719, R29720, R29721, R29722, R29723, R29724, R29725, R29726, R29727, R29728, R29729, R29730, R29731, R29732, R29733, R29734, R29735, R29736, R29737, R29738, R29739, R29740, R29741, R29742

PCI ICD-9-CM Codes: 0066, 1755, 3601, 3602, 3605

© 2021 Wassie M et al. *JAMA Network Open.*

PCI ICD-10-CM Codes: 0270346, 027034Z, 0270356, 027035Z, 0270366, 027036Z, 0270376, 027037Z, 02703D6, 02703DZ, 02703E6, 02703EZ, 02703F6, 02703FZ, 02703G6, 02703GZ, 02703T6, 02703TZ, 02703Z6, 02703ZZ, 0270446, 027044Z, 0270456, 027045Z, 0270466, 027046Z, 0270476, 027047Z, 02704D6, 02704DZ, 02704E6, 02704EZ, 02704F6, 02704FZ, 02704G6, 02704GZ, 02704T6, 02704TZ, 02704Z6, 02704ZZ, 0271346, 027134Z, 0271356, 027135Z, 0271366, 027136Z, 0271376, 027137Z, 02713D6, 02713DZ, 02713E6, 02713EZ, 02713F6, 02713FZ, 02713G6, 02713GZ, 02713T6, 02713TZ, 02713Z6, 02713ZZ, 0271446, 027144Z, 0271456, 027145Z, 0271466, 027146Z, 0271476, 027147Z, 02714D6, 02714DZ, 02714E6, 02714EZ, 02714F6, 02714FZ, 02714G6, 02714GZ, 02714T6, 02714TZ, 02714Z6, 02714ZZ, 0272346, 027234Z, 0272356, 027235Z, 0272366, 027236Z, 0272376, 027237Z, 02723D6, 02723DZ, 02723E6, 02723EZ, 02723F6, 02723FZ, 02723G6, 02723GZ, 02723T6, 02723TZ, 02723Z6, 02723ZZ, 0272446, 027244Z, 0272456, 027245Z, 0272466, 027246Z, 0272476, 027247Z, 02724D6, 02724DZ, 02724E6, 02724EZ, 02724F6, 02724FZ, 02724G6, 02724GZ, 02724T6, 02724TZ, 02724Z6, 02724ZZ, 0273346, 027334Z, 0273356, 027335Z, 0273366, 027336Z, 0273376, 027337Z, 02733D6, 02733DZ, 02733E6, 02733EZ, 02733F6, 02733FZ, 02733G6, 02733GZ, 02733T6, 02733TZ, 02733Z6, 02733ZZ, 0273446, 027344Z, 0273456, 027345Z, 0273466, 027346Z, 0273476, 027347Z, 02734D6, 02734DZ, 02734E6, 02734EZ, 02734F6, 02734FZ, 02734G6, 02734GZ, 02734T6, 02734TZ, 02734Z6, 02734ZZ, 02C03Z6, 02C03ZZ, 02C04Z6, 02C04ZZ, 02C13Z6, 02C13ZZ, 02C14Z6, 02C14ZZ, 02C23Z6, 02C23ZZ, 02C24Z6, 02C24ZZ, 02C33Z6, 02C33ZZ, 02C34Z6, 02C34ZZ

© 2021 Wassie M et al. *JAMA Network Open.*

PCI CPT Codes: 92980, 92981, 92982, 92983, 92984, 92985, 92986, 92987, 92988, 92989, 92990, 92991, 92992, 92993, 92994, 92995, 92996

© 2021 Wassie M et al. *JAMA Network Open.*
